# Postharvest quality maintenance of wax apple and guava fruits by use of a fermented broth of an ε-poly-l-lysine-producing *Streptomyces* strain

**DOI:** 10.1371/journal.pone.0265457

**Published:** 2022-03-16

**Authors:** Jian-Ling Bai, Hui-Hui Wang, Ju-Mei Zhang, Qing-Ping Wu, Shu-Ping Mo, Ying-Long He, Shao-Quan Weng, Xiao-Juan Yang, Ci-Zhou Li

**Affiliations:** 1 Guangdong Provincial Key Laboratory of Microbial Safety and Health, State Key Laboratory of Applied Microbiology Southern China, Institute of Microbiology, Guangdong Academy of Sciences, Guangzhou, China; 2 Guangzhou Wanglaoji Great Health Industry Co. Ltd., Guangzhou, China; Bahauddin Zakariya University, PAKISTAN

## Abstract

ε-Poly-l-lysine (ε-PL) is a natural antimicrobial polymer with significant inhibitory activity against a broad spectrum of microorganisms, and nowadays used widely as a preservative in the food industry. In the present study, ε-PL broth was obtained from *Streptomyces ahygroscopicus* GIM8 fermentation in a nutrient-limited liquid medium. The in vitro antifungal activity of the broth against fruit pathogens *Penicillium expansum* and *Colletotrichum gloeosporioides* was investigated, and its usage for postharvest storage of two highly perishable fruits wax apple and guava was evaluated. Results showed that ε-PL concentration in the broth reached 0.61 g/L, and the nutrition level of the broth was low. The antifungal activity of ε-PL broth was comparable to that of the aqueous solution of ε-PL under the same concentration. Immersion with the diluted broth (200 mg/L ε-PL) markedly delayed the decline in the quality of postharvest wax apple and guava fruits during storage, and the decay incidences were also greatly decreased as compared to their respective controls (distilled water immersion). A further investigation demonstrated that the ε-PL broth immersion induced an increase in the activity of defense-related enzymes peroxidase and polyphenol oxidase in the two fruits during storage. The present study proved that the fermentation broth of ε-PL could be used as a promising alternative to high purity ε-PL and synthetic fungicides for preserving fruits at postharvest stage.

## Introduction

Wax apple (*Syzygium samarangense* L.) is an important non-climacteric subtropical fruit, and planted widely in Southeast Asian countries including Thailand, Indonesia, and Malaysia [1]. Wax apple contains high levels of vitamin C and bioactive components, while its storage life is short (less than one week) under refrigerated storage condition due to its high respiration rate, flimsy skin, and high chilling sensitivity [2, 3]. Guava (*Psidium guajava* L.), another economically important fruit, has abundant biologically active substances, but it cannot be stored over three days after harvest at ambient condition because of fast ripening, abrupt softening, fungal attack, and high sensitivity to chilling [4, 5]. The anthracnose caused by *Colletotrichum* is a common fungal disease of postharvest guava [6]. Thus, there is an emerging demand for the development of effective technologies for postharvest storage of wax apple and guava fruits.

Over the past several decades, the use of chemical fungicides still remained the major way to control postharvest decay in fruits and vegetables. However, excessive use of fungicides results in drug resistance by pathogens [7], and chemical fungicides are harmful to human healthy as well as the environment [8]. Therefore, an increasing number of regulatory restrictions are put on the use of chemical fungicides. Because of these limitations, there is an urgent demand to develop alternative approaches to reduce or even replace chemical fungicides for postharvest storage of fruits and vegetables. Promising approaches, including natural antimicrobials like chitosan [9], natamycin [10], and tea oil [11], GRAS (generally regarded as safe) substances like trisodium phosphate [12], and biological control agents [13, 14], and elicitors [15] have been explored for the storage of postharvest fruits.

ε-Poly-l-lysine (ε-PL) is a cationic homopolyamide consisting of 25–30 l-lysine residues characterized by unique linkages between the ɛ-amino and α-carboxyl groups of l-lysine. It was first identified in culture filtrate of a *Streptomyces albulus* strain during screening of Dragendorff-positive substances [16], and currently produced at a commercial scale via aerobic fermentation in Japan and China. Due to its polycationic nature, ε-PL exhibits a broad-spectrum antimicrobial activity against bacteria, fungi, and yeasts, as well as some viruses [17, 18]. Besides the antimicrobial activity, ε-PL is degradable, thermally stable, and water soluble, and has non-toxicity to humans and the environment [19, 20]. Owing to the above-mentioned attributes, ε-PL is considered as a promising natural antimicrobial agent in the food industry. It was awarded GRAS status by the United States Food and Drug Administration [21]. Nowadays, ε-PL as a food preservative is used widely in a number of countries such as Japan, South Korea, the United States, and China [22].

Recently, ε-PL has attracted considerable attention for storage of postharvest fruits. Several studies showed that the fungal pathogens such as *Penicillium digitatum* and *Botrytis cinerea* isolated from fruits can be effectively inhibited by ε-PL [23, 24]. To the best of our knowledge, most studies initiated so far have concentrated on the use of high purity ε-PL for controlling postharvest diseases of fruits. Apparently, the cost was high, which is a major hindrance for its practical application in the fruit industry.

In the present study, considering the high price of ε-PL, the use of broth derived from ε-PL fermentation for postharvest storage of fruits was evaluated. The broth was typically prepared from submerged fermentation of an ε-PL-producing *Streptomyces ahygroscopicus* GIM8 in a medium with controlled nutrient levels. The antifungal activity of the broth against fruit pathogens was investigated, and then its postharvest use was assessed for preserving postharvest wax apple and guava fruits. The present study proved that the ε-PL broth could be a promising alternative to high purity ε-PL and chemical fungicides for postharvest storage of fruits. The use of ε-PL broth for fruit quality maintenance holds great promise for practical application in the fruit industry.

## Materials and methods

### Fruits

Mature fruits (either wax apple or guava) were harvested from a block with area less than 100 m^2^ in a commercial orchard in Conghua district, Guangzhou, Guangdong Province, China, and transported to the laboratory within 2 h. The fruit of uniform shape, size, and color and with no mechanical injuries or diseases were selected. The color of wax apple was pink, with L* value of 37.7±1.6 and a* value of 18.0±1.1. Guava had round shape and a bright color with L* value of 48.4±2.0 and a* value of 12.0±1.5. The initial firmness of wax apple and guava was 9.26±0.21 N and 12.17±0.18 N, respectively. Prior to use, the selected fruits were soaked in 0.5% sodium hypochlorite solution for 2 min, washed with tap water, and air-dried. For each fruit species, the selected fruits were randomly distributed into groups, with 30 fruits per group.

### Pathogens

The pathogens *Penicillium expansum* and *Colletotrichum gloeosporioides* were used in this work for the antifungal activity assay. *P*. *expansum* was isolated from an infected wax apple. *C*. *gloeosporioides* was an isolate of diseased guava fruit. Each pathogen was cultured on potato dextrose agar (PDA) medium in Petri dishes (9 cm diameter) at 25°C. After one week of incubation, spores of each of the pathogens were isolated by scraping the surface of the PDA medium with an inoculation loop, and suspended in 5 mL sterile distilled water containing 0.05% Tween 20 to prevent spore aggregation. To remove any adhering mycelia, the spore suspensions were filtered through four lagers of cheesecloth. The concentration of spores in the suspensions was counted using a hemocytometer, and adjusted to 1.0×10^5^ spores/mL with sterile distilled water. The final spore suspensions were used in subsequent experiments.

### Strain, media, and ε-PL fermentation

An ε-PL-producing strain *S*. *ahygroscopicus* GIM8, isolated previously in our group [25], was used. It has been stored at the China Center for Type Culture Collection with collection number CCTCC M2011191.

A nutrient-limited medium, modified based on M3G medium [26], was used, and referred to as MM3G medium in this work. The MM3G medium was comprised of the following ingredients: 18 g glucose, 2 g yeast extract, 4 g (NH_4_)_2_SO_4_, 1.36 g KH_2_PO_4_, 0.8 g K_2_HPO_4_, 0.5 g MgSO_4_·7H_2_O, 0.04 g ZnSO_4_·7H_2_O, 0.03 g FeSO_4_·7H_2_O, and 1000 mL distilled water. For sterilization, the medium was autoclaved at 121°C for 20 min.

For the preparation of seed culture, a loop of spores from a slant of a 5-day-old culture of *S*. *ahygroscopicus* GIM8 were inoculated into 30 mL MM3G medium in a 250 mL shake flask, and cultured at 30°C and 190 rpm for 20 h. For ε-PL fermentation, the seed culture (5%, v/v) was inoculated into 50 mL MM3G medium in a 250 mL flask, and incubation at 30°C and 190 rpm for a period of 72 h.

### Preparation of ε-PL broth

After a 72-h period of fermentation, the mycelia were removed by centrifugation at 8000×*g* for 10 min. The concentration of ε-PL in the resulting supernatants was then determined. The supernatants were diluted with sterile distilled water to required concentrations of ε-PL, and adjusted to neutrality (pH 7.0) using 1 M NaOH for better antimicrobial activity. The diluted ε-PL broth was used in subsequent experiments.

### In vitro antifungal activity assay

An aliquot (100 μL) of the spore suspensions was spread evently on the surface of PDA plates, and allowed to adsorb excess water into the medium for 2 h. Sterilized Oxford cups (7.8 mm in diameter) were placed in the center of a Petri dish (9 cm diameter), and then 50 μL of the diluted broth with ε-PL at a concentration range of 50 to 250 mg/L was pipetted into each cup. Distilled water was used as negative control, and the aqueous solution of ε-PL (powder, purity 99%, Zhejiang Silver Elephant Bioengineering Co., Ltd., Zhejiang Province, China) acted as positive control. The plates were incubated for 7 days at 25°C. For antifungal activity estimation, the diameter of inhibition zone was measured with a cross method using a caliper, and expressed as mm by subtracting the diameter of the cup from the measured values.

### Treatments

The fruits were immersed in the diluted broth (200 mg/L ε-PL) for 2 min. This concentration level of ε-PL was chosen based on previous published papers [23, 24] and our preliminary studies. For comparison, sterilized distilled water was used as negative control, and the ε-PL (200 mg/L) aqueous solution acted as positive control. Each treatment included three replicates, with 30 fruits per replicate. After immersion, the fruits of different treatments were separately placed in cartons with holes for aeration, and stored at 30°C and 80% relative humidity. During storage, physicochemical quality parameters of stored fruits were determined. The decay incidence of each treatment was recorded daily.

### Measurement of fermentation parameters

For biomass estimation, 10 mL of broth was filtered through a pre-weighted filter paper, washed, and dried at 80°C till a constant weight. Residual glucose levels in broth were measured using the 3,5-dinitrosalicylic acid colorimetric method. ε-PL was analyzed with a colorimetric method according to the procedures of Itzhaki [27]. The Bradford assay was used to quantify proteins with bovine serum albumin as the standard [28]. The exopolysaccharides were separated by ethanol precipitation, re-dissolved with distilled water, and then determined by the phenol-sulfuric acid method [29]. The pH of broth was measured with a pH meter.

### Determination of fruit quality parameters

Weight loss of fruits as percentage (%) was calculated by the following formula: (A-B)/A × 100, where A is the initial fruit weight, and B is the fruit weight after a certain storage period.

Total soluble solids (TSS) content in fruit flesh was determined by a portable refractometer (PAL-1, Atago, Tokyo, Japan). For each treatment, five fruits of each replicate were homogenized and mixed together. The obtained fruit juice was used to determine the TSS level. The result was expressed as percentage on a fresh weight (FW) basis.

Titratable acidity (TA) in fruit flesh was measured using the titration method [30]. Five fruits of each replicate in each treatment were homogenized by adding appropriate amount of distilled water and filtered. An aliquot of 20 mL was titrated against 0.1 M NaOH. The results were represented as the percentage of citric acid on a FW basis.

Ascorbic acid content determination was based on the method described by Terada et al. [31]. Five grams of tissue were obtained from five fruits (as above), and homogenized with 20 mL of cold 6% metaphosphoric acid, and centrifuged for 10 min at 12000×*g* and 4°C. A 1.6 mL of supernatants was mixed with 0.8 mL 2% di-indophenol, and then 1.6 mL 2% thiourea and 0.4 mL 1% dinitrophenol hydrazine were added. The mixture was incubated at 37°C for 3 h, and then 2.0 mL 85% H_2_SO_4_ was added, and against left at ambient temperature for at least 30 min. The absorbance at 540 nm was recorded. The ascorbic acid content was calculated as mg per 100 g FW.

### Assays of enzyme activity

The fruit samples were peeled. Ten grams of fruit tissue from five fruits of each replicate in each treatment was homogenized in 20 mL 0.05 M phosphate buffer (pH 7.8) at 4°C. The homogenate was centrifuged for 10 min at 10000×*g* and 4°C. The supernatants were used as enzyme extract for the analysis of activities of peroxidase (POD) and polyphenol oxidase (PPO).

The enzyme activity of POD in fruits was determined as previously described [32]. The reaction system was composed of 2.2 mL 1% guaiacol, 0.2 mL 1.5% hydrogen peroxide, and 0.5 mL enzyme extract. Absorbance was measured using a UV-Vis spectrophotometer at 470 nm for 3 min. One unit of PPO activity was defined as the amount of enzyme which caused a change of 0.01 in absorbance per minute at 470 nm, and expressed as units per gram FW.

PPO activity in fruits was assayed according to the method of Fang et al. [33]. The reaction mixture consisted of 3 mL 0.01 M catechol and 0.5 mL enzyme extract. Absorbance was measured at 420 nm for 3 min. One unit of PPO activity was defined as the amount of enzyme which caused a change of 0.01 in absorbance per minute at 420 nm. The activity was presented as units per gram FW.

### Statistical analysis

All data are presented as the mean ± standard deviation of three replicates. The data were analyzed using one-way analysis of variance. Significant differences between means were determined using Duncan’s multiple range tests, and a *P* value of less then 0.05 was considered statistically significant.

## Results

### Performance parameters of ε-PL fermentation

As shown in Table 1, the ε-PL yield reached 0.61 g/L, the broth was acidic with a final pH value of 3.12, and the biomass was 4.27 g/L. With regard to nutrient components, the protein concentration was low at 6.04 mg/L, and the carbohydrates including glucose and exopolysaccharides were not detectable. In summary, the broth contained considerable amounts of ε-PL while a little nutrition.

**Table 1:** Data on ε-PL fermentation in flasks by *S*. *ahygroscopicus* GIM8.

Parameters		Parameters	
ε-PL (g/L)	0.61±0.05	Glucose (g/L)	0
Biomass (g/L)	4.27±0.26	Proteins (mg/L)	6.04±0.42
Final pH	3.12±0.23	Exopolysaccharides (g/L)	0

Values were measured at the end of fermentation; data represents as the mean ± standard deviation (n = 3).

### In vitro antifungal activity of ε-PL broth

**Fig 1:**
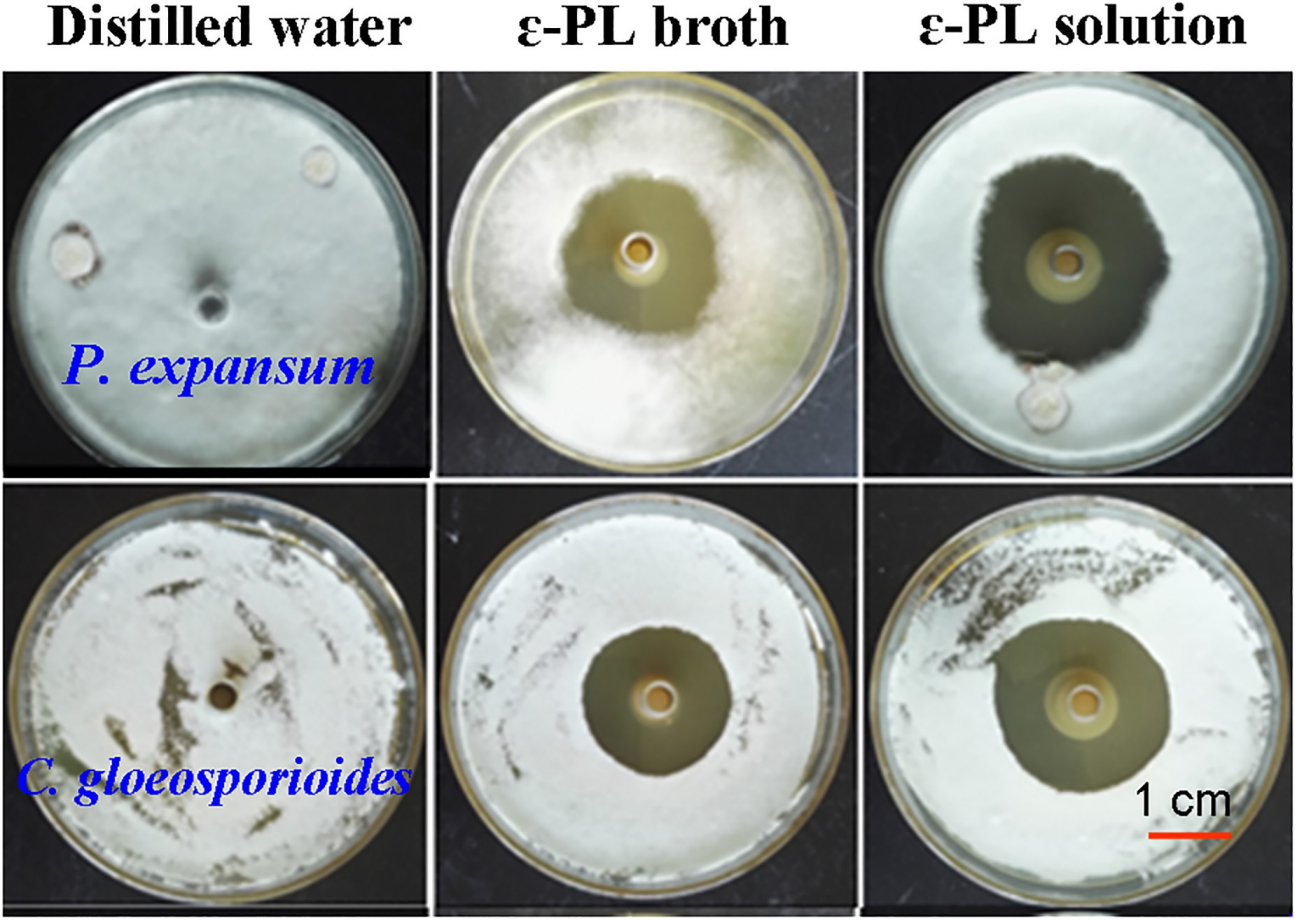
The growth of *P*. *expansum* and *C*. *gloeosporioides* in response to the broth and ɛ-PL solution in the PDA plates. ɛ-PL concentration was 200 mg/L in both solutions.

Fig 1 shows the inhibitory effect of the broth against *P*. *expansum* and *C*. *gloeosporioides*. The data on inhibition zone with different ε-PL levels (50–250 mg/L) are listed in Table 2. According to Fig 1, the diluted broth with 200 mg/L ε-PL had apparent inhibitory effect on the growth of the two pathogens, and the efficacy was comparable to that of the ε-PL solution under the same ε-PL concentration. Table 2 indicates that ε-PL at 50 mg/L in the broth or in distilled water did not show any inhibitory effect against the pathogens, while a weak effect could be observed as its concentration increased to 100 mg/L. At 150 mg/L ε-PL or above, the inhibition zone became larger, indicating strong antifungal activity. There were no significant differences in activity between the broth and ε-PL solution when the same ε-PL level was tested. The antifungal activity of the broth against other test fungi is shown in S1 Fig.

**Table 2:** In vitro antifungal activity of the broth and ε-PL solution against *P*. *expansum* and *C*. *gloeosporioides*.

ε-PL (mg/L)	Inhibitory zone (mm)			
	*P*. *expansum*		*C*. *gloeosporioides*	
	ε-PL broth	ε-PL solution	ε-PL broth	ε-PL solution
50	0.00±0.00a	0.00±0.00a	0.00±0.00a	0.00±0.00a
100	3.34±0.16a	3.36±0.14a	3.01±0.09a	3.05±0.11a
150	12.42±0.45a	12.65±1.15a	9.78±0.32b	10.11±0.45a
200	18.71±1.23b	19.02±1.06a	14.26±1.04a	14.44±0.82a
250	20.25±1.06a	20.08±1.32a	15.33±0.78a	15.67±1.20a

Values followed by the same letters within the same row for each strain are not significantly different (*P* < 0.05); data represents as the mean ± standard deviation (n = 3).

### Effects of ε-PL broth on weight loss of postharvest wax apple and guava fruits

**Fig 2:**
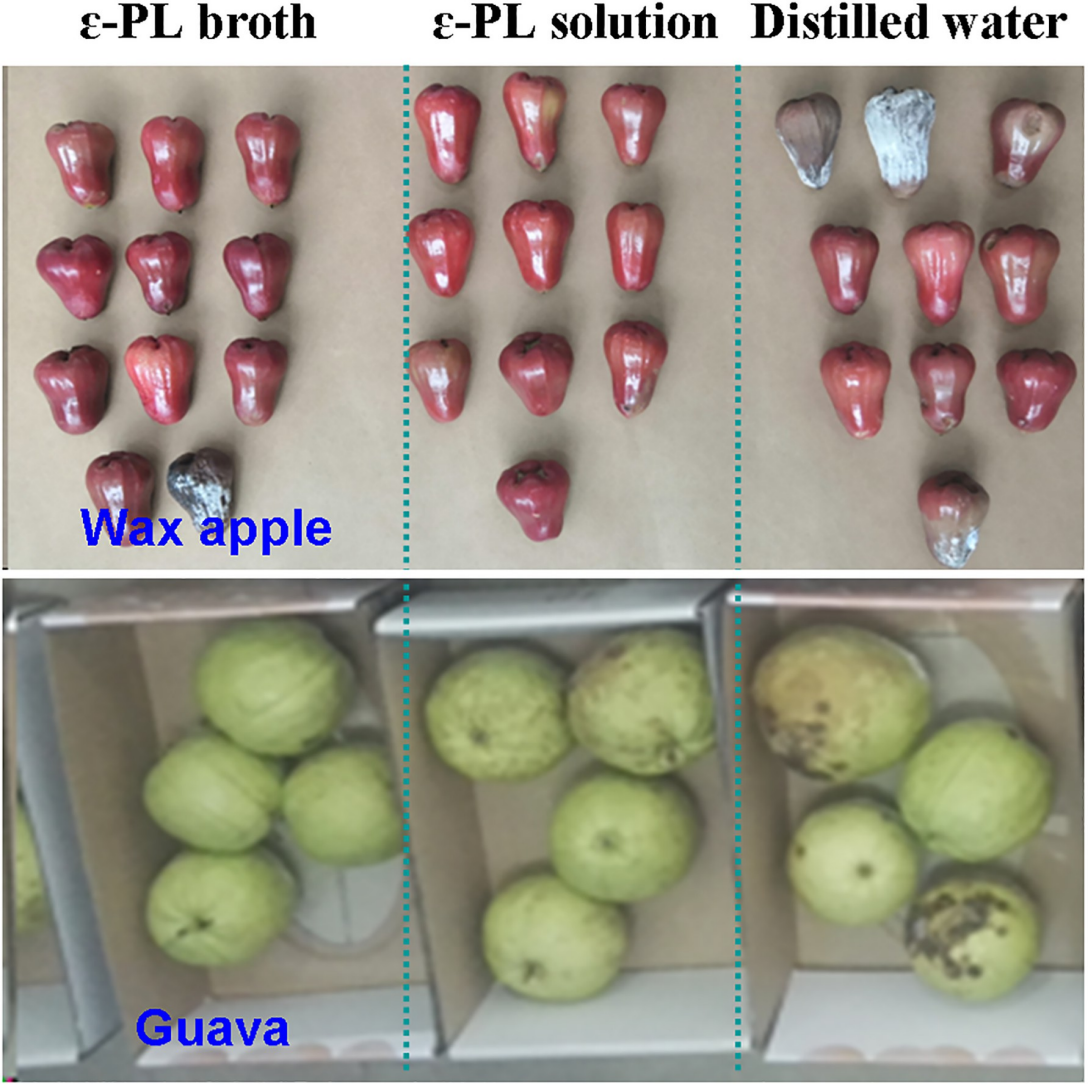
Visual appearance of wax apple and guava fruits with different treatments after a storage period of 7 days. ɛ-PL concentration was 200 mg/L in both solutions.

**Fig 3:**
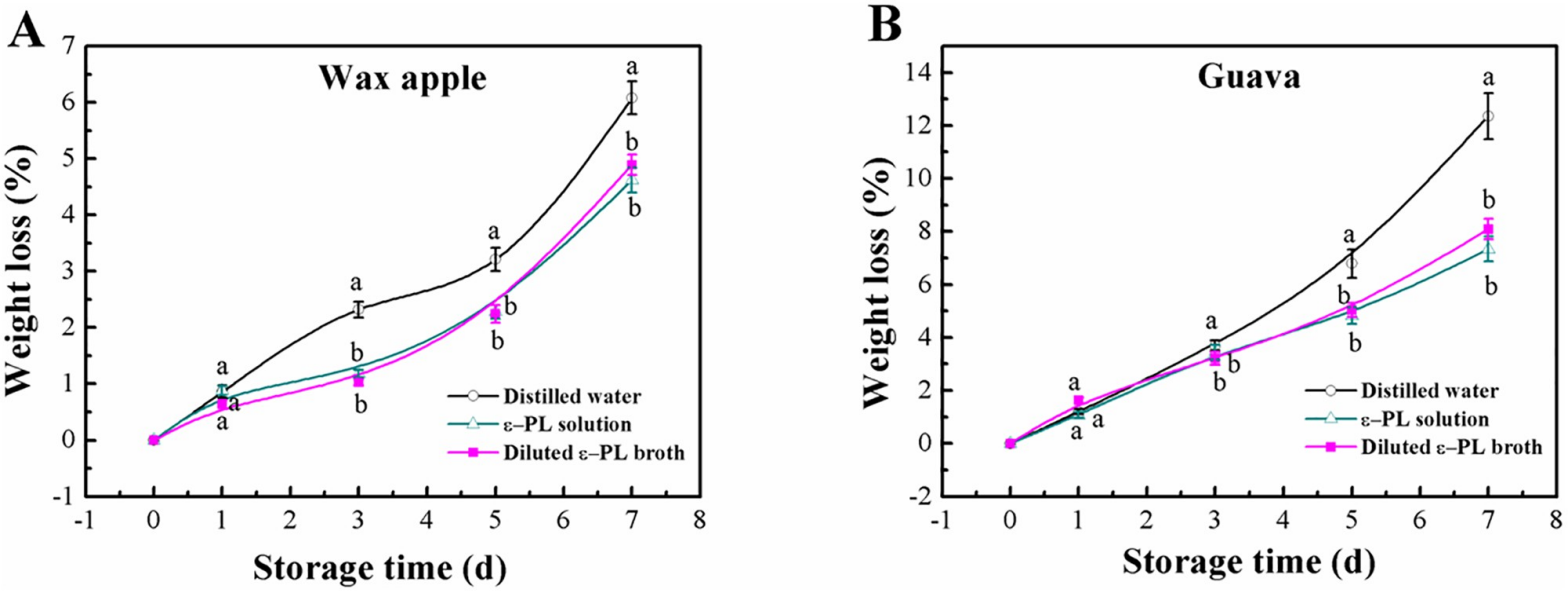
Changes in weight loss of wax apple and guava fruits with different treatments during postharvest storage. Different letters at the same time point indicate significant differences at *P* < 0.05. Vertical bars represent standard deviations of the means (n = 3).

Fig 2 shows the visual appearance of wax apple and guava fruits in the experimental and control groups after 7 days of storage. Clearly, the broth had similar effect to that of the ε-PL solution for the storage of the two fruits. As illustrated in Fig 3, the weight loss increased continuously with time during the entire storage period for all treatments, regardless of fruit species. Compared with the negative controls, the ε-PL broth markedly decreased weight loss of both tested fruits, and the retention effect was similar between the broth and the ε-PL solution. At the end of storage, the weight loss in the negative control wax apple fruit was 6.02%, while lower values of 4.89% and 4.62% were recorded in the broth- and ε-PL solution-treated groups, respectively. Similar results were obtained when guava fruits were used. Statistical analysis showed that interaction of treatment and storage time was not significant on weight loss of both fruits, while main effect of the two factors found significant.

### Effects of ε-PL broth on TSS, TA, and ascorbic acid contents

As shown in Table 3, the TSS content of wax apple with distilled water immersion first rose and then decreased rapidly from 3 days of storage. Differently, a gradual increase was observed throughout the storage period when the broth or ε-PL solution was used. No significant differences in TSS content of wax apple were observed between the broth and ε-PL solution immersion in all assayed time points, but the differences were significant between the broth or ε-PL solution immersion with the distilled water immersion. From Table 4, there was a gradual increase of TSS content in guava fruit during storage for all treatments. Compared to the distilled water, both the broth and ε-PL solution retarded the increase in TSS content, and the difference in inhibition effect was minor between the broth and ε-PL solution.

**Table 3:** TTS, TA, and ascorbic acid contents of wax apple at different storage time with different treatments.

Time (d)	TTS (%)			TA (%)			Ascorbic acid (mg/100 g)		
	ɛ-PL broth	ɛ-PL solution	Distilled water	ɛ-PL broth	ɛ-PL solution	Distilled water	ɛ-PL broth	ɛ-PL solution	Distilled water
0	5.40±0.17a	5.40±0.17a	5.40±0.17a	0.31±0.02a	0.31±0.02a	0.31±0.02a	6.83±0.41a	6.83±0.41a	6.83±0.41a
1	5.44±0.13a	5.46±0.23a	5.45±0.18a	0.29±0.03a	0.30±0.02a	0.28±0.02a	5.77±0.26b	6.09±0.34a	4.91±0.24c
3	5.61±0.19a	5.58±0.21a	5.61±0.15a	0.25±0.02b	0.27±0.02a	0.23±0.01c	5.31±0.32a	5.53±0.23a	4.12±0.23b
5	5.87±0.21a	5.92±0.19a	5.81±0.22b	0.20±0.01a	0.21±0.03a	0.19±0.03a	4.35±0.23a	4.23±0.21a	3.21±0.21b
7	6.03±0.14a	6.11±0.12a	5.17±0.21b	0.17±0.02a	0.18±0.02a	0.14±0.02b	3.24±0.17a	3.28±0.24a	2.46±0.14b

ɛ-PL concentration was 200 mg/L in the broth and ɛ-PL solution; values followed by the same letters within the same row for each quality parameter are not significantly different (*P* < 0.05); data represents as the mean ± standard deviation (n = 3).

**Table 4:** TTS, TA, and ascorbic acid contents of guava at different storage time with different treatments.

Time (d)	TTS (%)			TA (%)			Ascorbic acid (mg/100 g)		
	ɛ-PL broth	ɛ-PL solution	Distilled water	ɛ-PL broth	ɛ-PL solution	Distilled water	ɛ-PL broth	ɛ-PL solution	Distilled water
0	8.20±0.34a	8.20±0.34a	8.20±0.34a	0.35±0.02a	0.35±0.02a	0.35±0.02a	83.21±3.84a	83.21±3.84a	83.21±3.84a
1	8.24±0.23b	8.21±0.18b	8.43 ±0.22a	0.33±0.03a	0.34±0.02a	0.31±0.03a	71.38±3.16a	73.26±3.24a	72.65±3.52a
3	8.42±0.32b	8.31±0.21b	8.87±0.24a	0.27±0.02b	0.29±0.01a	0.25±0.02c	58.46±2.78b	61.10±3.27a	55.60±2.47a
5	8.53±0.25b	8.43±0.26b	9.12 ±0.35a	0.24±0.03b	0.28±0.02a	0.20±0.03c	50.65±2.88a	53.45±3.14a	42.10±2.34b
7	8.74±0.22bc	8.51±0.23c	9.52±0.28a	0.18±0.01b	0.21±0.03a	0.14±0.01c	46.44±2.54a	46.51±2.22a	36.47±1.98b

ɛ-PL concentration was 200 mg/L in the broth and ɛ-PL solution; values followed by the same letters within the same row for each quality parameter are not significantly different (*P* < 0.05); data represents as the mean ± standard deviation (n = 3).

TA of wax apple and guava fruits in all test groups reduced over storage time, while the reductions were greater in the negative control groups as compared with their corresponding experimental and positive control groups (Tables 3 and 4). Significant differences in TA level of each test fruit could be readily observed between the negative control group with the experimental group, or with the positive control group. The broth and ε-PL solution had similar effect in delaying TA reduction in wax apple, but a less effect was showed by the broth compared to the ε-PL solution in the case of guava fruit.

The ascorbic acid content in both wax apple and guava fruits decreased continuously with storage time in all treatments (Tables 3 and 4). Compared with the distilled water immersion, the broth retarded the decline of ascorbic acid content in wax apple. This phenomenon was also observed when using the ε-PL solution. In the case of guava fruit, the broth showed a less retention effect in preventing ascorbic acid degradation compared to the ε-PL solution, but there were no significant differences except at 3 days of treatment.

### Effects of ε-PL broth on the decay incidence

**Fig 4:**
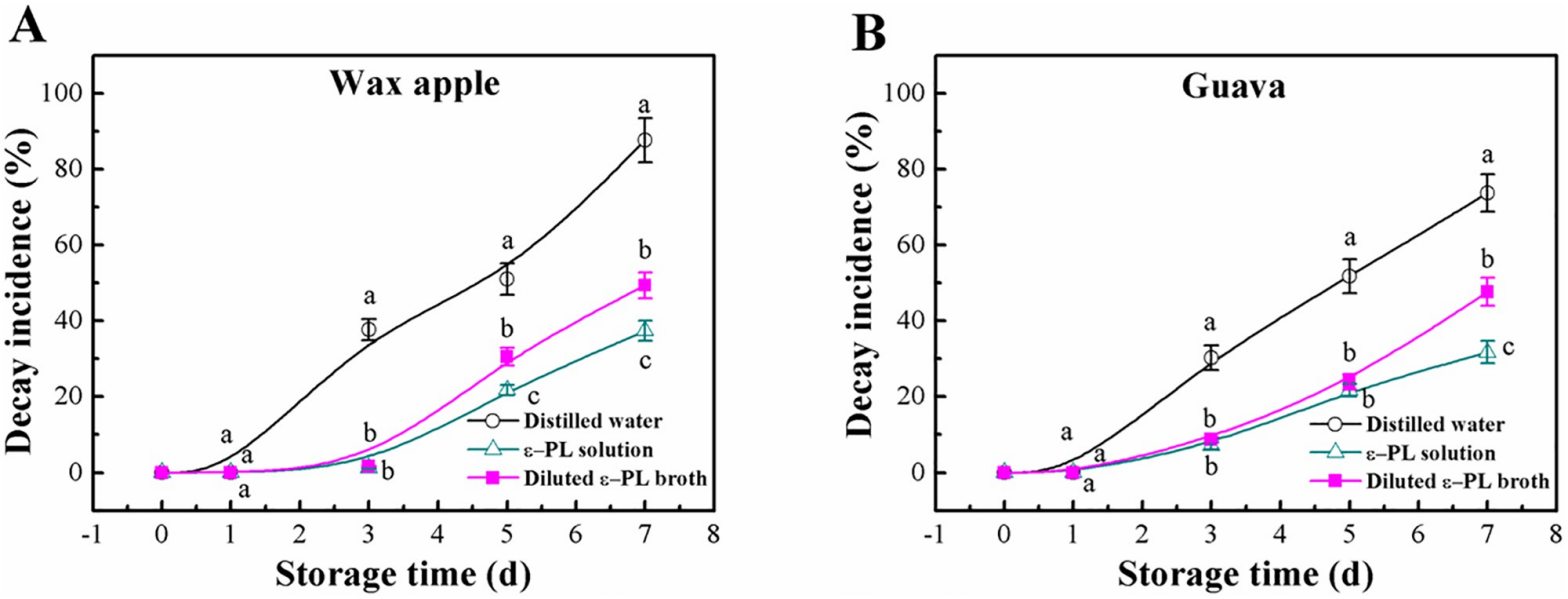
Effects of ε-PL broth on decay incidence of wax apple and guava fruits during a 7-day period of storage. ε-PL concentration was 200 mg/L in both solutions. Different letters at the same time point indicate significant differences at *P* < 0.05. Vertical bars represent standard deviations of the means (n = 3).

Fig 4 shows that the decay incidence of both wax apple and guava fruits increased continuously with storage time for all treatments. In the negative control wax apple fruit, the decay incidence increased rapidly to 37.74% at 3 days of storage, while the fruit in the experimental and positive control groups were still not infected (Fig 4A). After a storage period of 7 days, the decay incidence of wax apple in the negative control reached as high as 87.64%, while lower values at 49.35% and 37.36% were recorded for the broth and ε-PL treatments, respectively. For guava fruit, Fig 4B shows that the broth exhibited effects similar to that of the ε-PL solution in inhibiting fungal decay. After 7 days of storage, the decay incidences were 73.75%, 47.66%, and 31.73% for the distilled water, the broth and ε-PL solution treatments, respectively. The statistical analysis showed that interaction of treatment and storage time was not significant on decay incidence of both fruits, while main effect of the two factors found significant.

### Effects of ε-PL broth on the activity of defense-related enzymes

**Fig 5:**
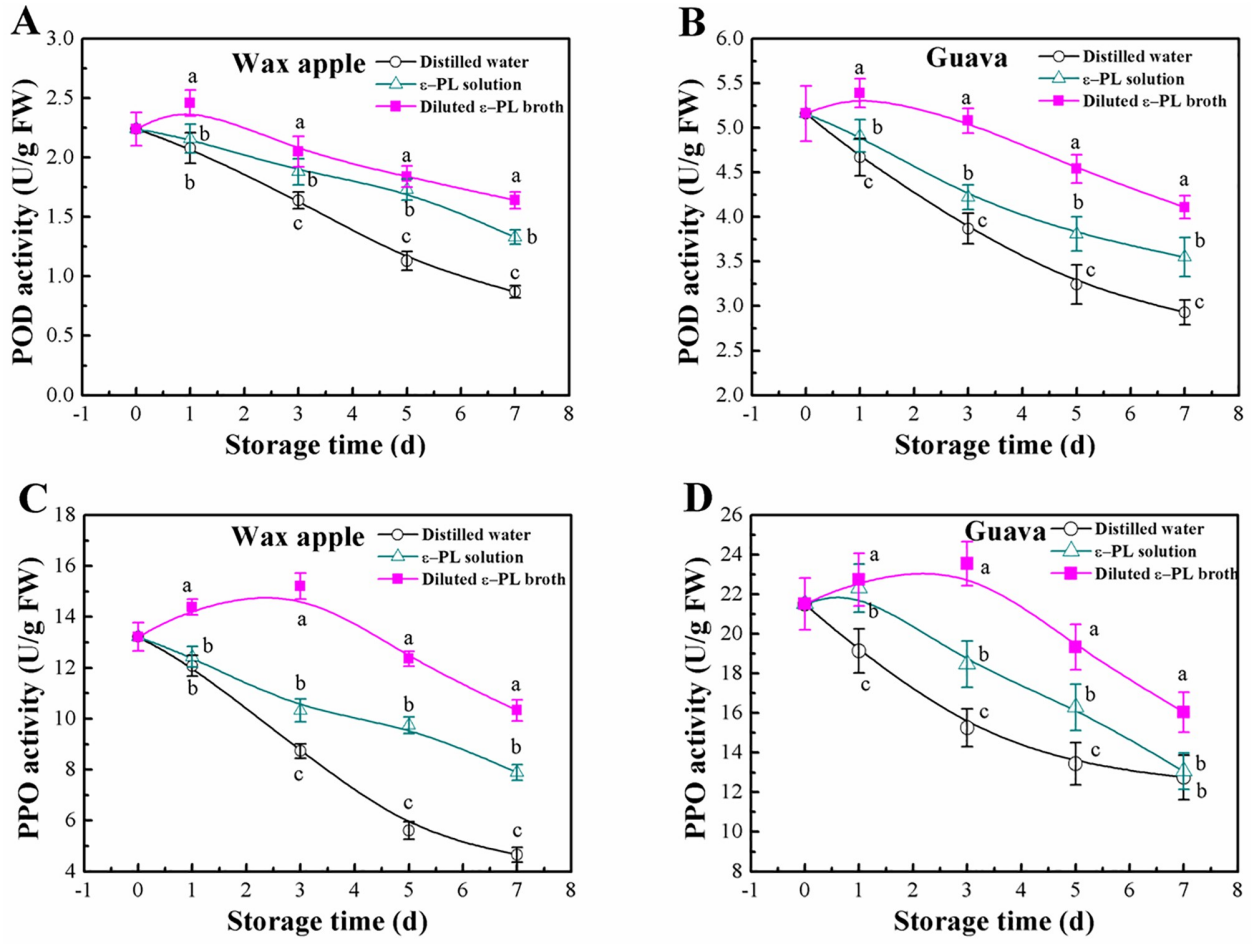
Effects of ε-PL broth on the activity of POD and PPO in wax apple and guava fruits during postharvest storage. ε-PL concentration was 200 mg/L in both solutions. Different letters at the same time point indicate significant differences at *P* < 0.05. Vertical bars represent standard deviations of the means (n = 3).

As observed in Fig 5, POD activity of both wax apple and guava fruits treated with distilled water or ε-PL solution showed a decreasing trend throughout the storage period. Differently, the ε-PL broth immersion induced an increase of POD activity in both fruits on day 1, afterwards it decreased continuously with time. POD activity in both kinds of fruits with the broth or ε-PL solution immersion was significantly higher than those with distilled water. In both test fruits, the improvement in POD activity was higher by the broth induction than by the ε-PL solution induction, and the differences were statistically significant from one day of treatment up to the end of storage period.

Results in Fig 5 showed that the activity of PPO in both wax apple and guava fruits in the negative and positive controls decreased continuously with time, while the reduction was greater in the negative control fruits compared to the corresponding positive control fruits. Contrary to this, with the ε-PL broth, PPO activity in the two tested fruits increased gradually, peaking at 3 days of storage, and then decreased continuously. Of the three treatments, with the broth treatment, the activity of PPO in each test fruit was highest after one day up to the end of storage, and the lowest activity was shown in the distilled water treatment.

## Discussion

As a natural and safe antimicrobial agent, ε-PL has been used to control fungal diseases of harvested fruits such as jujube fruit and citrus fruit [23, 24]. ε-PL also demonstrated the potential to maintain quality and extend shelf life of fresh-cut fruits and vegetables [34, 35]. Among these investigations, the commercial ε-PL with relatively high purity in a powder form was used. However, the market price of ε-PL was high, reaching 2.0×10^5^ USD/metric ton [36], hence, its practical application is not economically viable in the fruit industry.

The highest yield of ε-PL to date reached a level of 59.5 g/L in a fed-batch process using a high-yield mutant [37]. However, as the separation and purification of ε-PL from fermentation broth included flocculation, filtration, ultrafiltration, ion-exchange chromatography, and decoloration steps [38], the total production cost of ε-PL is still high. To reduce the cost of ε-PL usage in the fruit industry, the use of ε-PL broth may be a feasible solution. In the present study, a nutrient-limited medium was used for ε-PL fermentation and a broth with a low level of the major nutrients while having acceptable ε-PL titer was obtained.

The utilization of fermentation broth of various microbes has received more and more attention in recent years. In a study done by Sarnthima et al. [39], the culture broth of the medicinal mushroom *Ganoderma lucidum* was found to have strong antibacterial and antioxidant activities, and the authors pointed out that it could be used as a value-added ingredient in the products such as cosmetics and nutraceuticals. A broth was obtained through fermentation of Chinese herbs with probiotics, and showed the potential to store postharvest citrus fruit [13]. Ma et al. [40] demonstrated that the pre-harvest treatment of kiwifruit trees with mixed culture fermentation broth of *Trichoderma pseudokoningii* and *Rhizopus nigricans* prolonged the shelf life and improved the quality of postharvest fruit.

In the present study, the ε-PL broth was tested to maintain the quality of subtropical fruits wax apple and guava during postharvest storage. The weight loss of wax apple and guava increased continuously with time in all treatments. Loss of weight in fresh fruits and vegetables is mainly due to loss of water caused by transpiration and respiration processes [41]. Interestingly, the broth and ε-PL aqueous solution immersion retarded the decline in weight loss of both test fruits compared to their respective negative controls. This can be explained by high water absorbency of ε-PL. The increase of TTS content in fruits is suggested to be related to the hydrolysis of starch and other macromolecular substances [42], and the conversion of organic acids to sugars and moisture loss by the fruits [43]. A lower increase rate of TTS in the fruits treated with the broth and ε-PL solution was found in this work, as compared with the distilled water treatment. This may be due to the inhibition of respiration of the fruits by ε-PL.

Ascorbic acid is an important antioxidant in fruits. It has been suggested that the ascorbic acid content loss in postharvest fruits was related to the O_2_, pH, and other factors [34]. In the present study, the ascorbic acid retention rates of postharvest wax apple and guava fruits with the broth and ε-PL solution treatments were higher than those of their respective negative control fruits. The reason for this can be attributed to a lower respiration rate in the fruits due to the action of ε-PL.

Biological control with antagonistic microorganisms has been considered as the most promising strategy to control postharvest diseases of fruits [44]. A great number of antagonistic microorganisms, such as yeasts, *Bacillus*, and *Streptomyces* had demonstrated the potential to prevent fungal decay during postharvest storage of fruits [14, 45, 46]. Also, the combined use of antagonistic microbes with preservatives generally presents a synergetic effect during storage of postharvest fruits [14, 47]. Several biocontrol mechanisms have been proposed, including competitions for space with pathogens, production of various bioactive substances with antibiotic activity and cell wall-degrading compounds, and induction of systemic resistance [48].

Inducing disease resistance is an important strategy to improve the degree to which plants and postharvest fruit use their own defense mechanisms against pathogens [47]. POD and PPO are important enzymes associated with disease resistance in plants, and involved in active oxygen metabolism in fruit and participate in the oxidation of phenolic compounds to toxic quinines [49]. In the present study, the activities of both POD and PPO in wax apple and guava fruits were induced by the ε-PL broth. The induction may be mainly due to the spores and a small amount of mycelial fragments present in the broth, which served as antagonistic microbes. We therefore suggested that in addition to the antifungal action of ε-PL, the induction of disease resistance by the ε-PL broth is another mechanism for its preservation effect.

## Conclusion

The present study demonstrated that the ε-PL broth, prepared from *S*. *ahygroscopicus* fermentation, had strong inhibitory effects against fungal pathogens of postharvest fruits. The ε-PL broth immersion delayed the decline in the fruit quality of harvested wax apple and guava fruits during storage, and induced disease resistance in the fruits. In conclusion, the ε-PL broth could be employed directly as an alternative to high purity ε-PL and other fungicides for postharvest storage of fruits.

## Supporting information

S1 Fig In vitro antifungal activity of ɛ-PL broth against *Pestalotiopsis microspora*, *Aspergillus flavus*, and *Penicillium verrucosum* in the PDA plates. ɛ-PL concentration was 200 mg/L in the broth and ɛ-PL solution.
(TIF)
