# Supplementary figures and images for: Postharvest quality maintenance of wax apple and guava fruits by use of a fermented broth of an ε-poly-l-lysine-producing Streptomyces strain
Source: PLoS One. 2022 Mar 16;17(3):e0265457. doi: 10.1371/journal.pone.0265457 (PMC8926194; doi:10.1371/journal.pone.0265457)

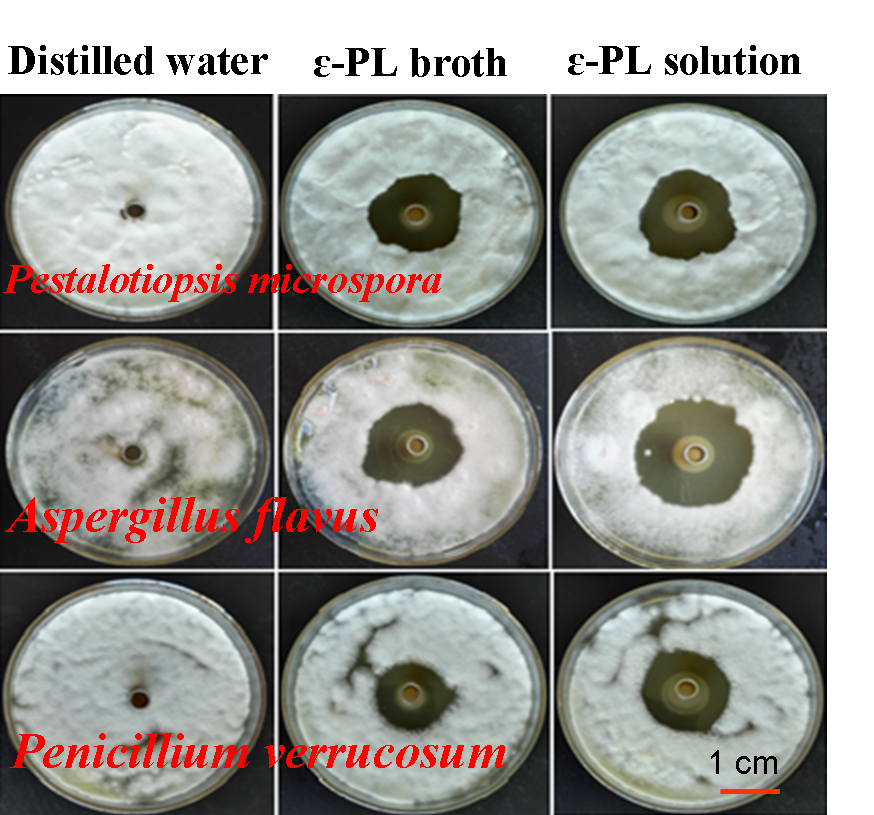

Supplement: S1 Fig — ɛ-PL concentration was 200 mg/L in the broth and ɛ-PL solution. (TIF) [file pone.0265457.s001.tif]
